# The Curated Food System: A Limiting Aspirational Vision of What Constitutes “Good” Food

**DOI:** 10.3390/ijerph17176157

**Published:** 2020-08-25

**Authors:** Lauri Andress, Carmen Byker Shanks, Annie Hardison-Moody, T. Elaine Prewitt, Paul Kinder, Lindsey Haynes-Maslow

**Affiliations:** 1 Department of Health Policy, Management, and Leadership, West Virginia University, 64 Medical Center Drive, Morgantown, WV 26506-9190, USA; 2 Department of Health and Human Development, Food and Health Lab, Montana State University, Bozeman, MT 59718, USA; 3 Department of Agricultural and Human Sciences, North Carolina State University, Raleigh, NC 27695, USA; amhardis@ncsu.edu (A.H.-M.); lhmaslow@ncsu.edu (L.H.-M.); 4 Department of Health Policy and Management, Fay W. Boozman College of Public Health, University of Arkansas for Medical Sciences, Little Rock, AR 72205, USA; tprewitt@uams.edu; 5 Natural Resource Analysis Center, West Virginia University, Morgantown, WV 26506, USA; Paul.Kinder@mail.wvu.edu

**Keywords:** rural, food system, inequities, disparities, food security

## Abstract

In an effort to elucidate an aspirational vision for the food system and explore whether the characteristics of such a system inadvertently set unattainable standards for low-wealth rural communities, we applied discourse analysis to the following qualitative datasets: (1) interviews with food experts and advocates, (2) scholarly and grey literature, (3) industry websites, and (4) email exchanges between food advocates. The analysis revealed eight aspirational food system discourses: production, distribution, and infrastructure; healthy, organic, local food; behavioral health and education; sustainability; finance and investment; hunger relief; demand-side preferences; romanticized, community led transformations. Study findings reveal that of eight discourses, only three encompass the experiences of low-wealth rural residents. This aspirational food system may aggravate the lack of autonomy and powerlessness already experienced by low-wealth rural groups, perpetuate a sense of failure by groups who will be unable to reach the aspirational food vision, silence discourses that might question those that play a role in the inequitable distribution of income while sanctioning discourses that focus on personal or community solutions, and leave out other policy-based solutions that address issues located within the food system. Further research might explore how to draw attention to silenced discourses on the needs and preferences of low-wealth rural populations to ensure that the policies and programs promoted by food system experts mitigate poor diets caused by food insecurity. Further research is needed to inform policies and programs to mitigate food insecurity in low-wealth rural populations.

## 1. Introduction

Several studies have noted that the issue of poor diets has been curated by food system experts and advocates into a problem whose solutions do not always address the needs of low-wealth groups [1,2]. This paper adds to these critical analytical perspectives by exploring how food system experts and advocates have created an aspirational vision of the food system where individuals gain higher social status by virtue of the decisions they make to eat “good” food that delivers a broad array of outcomes including equity, environmental sustainability, healthy food access, and community level economic benefits [3], to name just a few. The definition of what constitutes socially acceptable or “good” food in the food system, then, becomes a contested concept that requires examination through the discourses in the analysis that follows.

With the acceptance of this aspirational food system, we argue that food experts and advocates are in danger of establishing a food system that fails to acknowledge variations in social status, stigmatizes groups that cannot arrange their lives to conform to the specifics of the vision, and results in policies and programs that fail to address the problem of poor diets based on the food insecurity experienced by low-wealth rural populations. This paper builds upon the co-author’s qualitative research and experience working alongside low-wealth rural populations to argue that current food system discourses establish a set of aspirational goals that are curated to exclude everyday lived experiences of low-wealth rural individuals.

### 1.1. Key Concepts

The key concepts of “rural,” “curation,” “aspirational class,” “food systems,” and “discourse” are leveraged to investigate the basis for this study that today’s expert vision of the food system authenticates a contested image of “good” food that establishes aspirations more likely to discount the discourses, needs, and preferences of low-wealth rural groups.

#### 1.1.1. Rural

Driven by unemployment and underemployment, rural Americans are more likely to experience poverty than urban Americans [4]. Poverty contributes to food insecurity in rural areas, or a “lack of consistent access to enough food for an active, healthy life” [5]. Food insecurity rates are higher in rural compared to urban communities in the United States, with 12.7% of rural households reporting that they are food insecure compared to 10.8% of urban households [4]. These disparities, when coupled with potentially lower social status due to food choices and food access, have the potential to further marginalize low-wealth rural groups relative to groups able to make food choices that designate them as high social status.

#### 1.1.2. Curation

The concept of curate, from the Latin curare, means to care and was reflected in Ancient Rome as high-ranking civil servants who cared for vital infrastructure including bathhouses, aqueducts, and water/sewer systems [6]. The act of curating did not come into its own until the late 1960s when the curator as creator was born [7]. At that point in the history of art, museums, and exhibitions, curators became those who made intentional choices about what was relevant to their audiences [6]. Today, content about a specific concept beyond the art world, as in this case food and the food system, is often gathered, studied, labeled, and assembled by an expert to signify that there is a binding theme.

#### 1.1.3. Aspirational Class

In *The Sum of Small Things*, Elizabeth Currid-Halkett describes the aspirational vision as an updated version of the notion of conspicuous consumption [8,9]. Up until the late 20th century, social class was based on the consumption of hard-to-obtain, high-priced, physical goods such as purses, shoes, or automobiles. In contrast, as these hard-to-obtain goods have become easier to obtain due to mass production and falling prices, they have been replaced by aspirational consumption where social status is exhibited by more subtle, less visible choices made in the acquisition of experiences and knowledge (i.e., travel, memberships to a concert series, museums, art galleries, admission to specific educational institutions), certain foods (i.e., heirloom tomatoes, grain-fed beef, free-range chicken eggs, locally produced foods), healthy activities (i.e., yoga, Pilates), and lifestyle (i.e., parenting, nannies, breastfeeding, housekeepers) [8]. Operating through discreet, inconspicuous, and skillful decision making as opposed to prominent displays of wealth, the aspirational class is thought to reproduce a vision of social status and upward mobility that deepens a widening class divide in the U.S. [8].

#### 1.1.4. Food System

A popular version of the U.S. aspirational food system starts with food experts and advocates that have problematized the notion that average citizens lack a model that envisions food as part of a supply chain starting with production and ending with consumption [10]. This particular problematization of the food system by experts and advocates is intended to heighten awareness of a food supply chain that, unlike the modern food system, is closer to local or regional places built around the notion of a socially constructed local or regional community [11,12]. The goal of narratives around this food system is to equip citizens with a model and vision of a food supply chain that encompasses a number of related domains that might fall under a social justice paradigm including healthy nutrition, local agriculture, the environment, equity, and sustainability [13].

### 1.2. Discourse

Discourse, in its simplest form, may be described as passages of connected writing or speech. From a more critical perspective, discourse is described as a group of meaningful statements or texts that appear to have a common theme and have effects on society [14]. Passages of discourse do not typically reveal the truth or falseness of statements but may help in discerning the spatial, temporal, and social circumstances that privilege certain discourses [14]. Discourses that are dominant or more pervasive generally reveal who is empowered and dominant versus voices that are shut out.

In the case of this paper, discourse will be used to identify patterns of writing and speech that have been curated by food system experts and advocates resulting in aspirational ideals that dominate the voices of low-wealth rural citizens starting in the late 20th century and going forward.

The curatorial process of converting the food system into this aspirational vision is alarming because some of these “expert” ideas about “good” food involve expenditures that are out of reach for low-wealth rural individuals, which in turn exacerbates the already stark divide between groups who can get that food and those who cannot. Further, the curation of the food system into an aspirational vision ignores the preferences, lived experiences, knowledge, and strategies used by low-wealth rural people to secure food [15,16]. Such a vision has the potential to increase stigma and surveillance for those who are already socially marginalized for the inability to meet this aspirational vision. Finally, food is important and sets the stage for health and well-being of the current and next generation. Failure to recognize how hard it is to achieve the curated aspirational vision of food signifies insensitivity to the realities of the lived experiences of others who are unable to achieve that vision and a failure to create system structures and policies that promote health equity and social justice [8,15,16,17].

## 2. Materials and Methods 

### 2.1. Study Design

Data from scholarly and grey literature, websites, interviews with food system experts and advocates, and an email exchange between food system experts was used to identify discourses and passages that exemplified the discourses. In qualitative research, with respect to sample size, it is often sufficient to use a sample of just a few texts, documents, or interview transcripts because the focus of interest is on repetition until the saturation point is reached [18,19]. Understanding the “how” and “why” is elevated in qualitative research over “how many” [20,21]. In this case, the identification of patterns in language or ideas instead of people is key.

The email set of qualitative data was sourced from an exchange between food system experts that occurred in March and April 2020. Authors (L.A., C.B.S., A.H.-M., T.E.P., L.H.-M.) sent an email to ask these experts for input on a code book and themes to be used as part of a public health master’s level course in issue analysis and food systems.

The set of qualitative data from interviews with food system experts and advocates throughout 2019 was originally generated because of a project on food deserts in West Virginia. Snowball sampling by word of mouth was used to recruit food system experts and advocates [22]. A semi-structured interview guide with open-ended questions was used to conduct interviews from April to November 2019. The initial interview guide was developed by a researcher (LA) based on a review of documents from a 2018 food desert meeting at West Virginia University and public health and geography literature on food deserts. The interview guide was reviewed by several food system and agricultural experts in West Virginia. Interviews of approximately 60 min were recorded and professionally transcribed verbatim. A secondary analysis of the transcripts was undertaken to identify food systems discourses and passages that exemplified the discourses [23,24]. A demographic profile for the participants in the food desert interviews and email exchange are exhibited in Table 1. The collection of expert interview data was approved by the Institutional Review Board of West Virginia University.

### 2.2. Data Analysis

To identify evidence of aspirational food discourses, we used discourse analysis, which can shed light on how some statements, texts, and images become acceptable within a certain time and place and, in doing so, become dominant, common sense while simultaneously silencing (i.e., not a part of main stream discourse) other ideas, images, texts, and views [14].

The discourse analysis was undertaken consistent with tenets of grounded theory and an inductive approach to inquiry, where a theory is allowed to unfold and knowledge accrue as researchers are encouraged to focus data analysis less on the product of inquiry and more on methodological strategies that can handle problems that arise as inquiry proceeds [25]. To ensure that readers could understand the gathering and analysis of data and how its interpretation yielded the aspirational food discourses, the grounded theory methodological strategy selected was based on anthropologist Clifford Geertz’s conception of a “thick description”, where the researcher attempts to deepen understanding through sustained interaction and close attention to another culture or phenomenon [26].

Thick descriptions pay close attention to contextual detail in observing and interpreting social meaning when conducting qualitative research so that by describing a phenomenon in sufficient detail one can begin to evaluate the extent to which the conclusions drawn are transferable to other times, settings, situations, and people [27].

Because thick descriptions do not simply describe what is observed, the principal researcher for this study (L.A.) observing methodological directions, provided descriptive interpretations illustrating the significance, meaning, and motivation behind the qualitative datasets used for this study [28]. A second author (C.B.S.) reviewed the discourses and supporting descriptions and came to agreement with the principal researcher. The other co-authors (A.H.-M., T.E.P., P.K., L.H.-M.) provided tertiary input on the discourses and supporting descriptions, particularly from their expert experiences collecting food system data in rural areas of the U.S.

Our thick descriptions of the discourses included passages from the qualitative data along with narrative profiles. To aide in the development of narrative profiles, we created a set of screening questions adapted from frameworks of previously published examinations of discourses on the social determinants of health, obesity, and food security (Table 2) [16,29,30,31].

## 3. Results

The analysis revealed eight aspirational food system discourses: (1) production, distribution, and infrastructure; (2) healthy, organic, local food; (3) behavioral health and education; (4) sustainability; (5) finance and investment; (6) hunger relief; (7) demand-side preferences; and (8) romanticized, community led transformations. A summary of the thick descriptions and narrative profiles for each of these discourses may be found in Table 3.

### 3.1. Production, Distribution, and Infrastructure 

This discourse considers how food is grown, processed, transported, and generates a profit for local farmers in a rural state. The cognitive model most prevalent in this discourse starts with production defined as farmers. The discourse highlights the ability of local farmers to earn a living distributing their products [32]. A key idea furthered by this discourse is the importance of a robust supply chain that results in an income for local farmers. The narrative defines equity as a food chain that concludes when the farmer’s products are bought by a distributor at fair market value that allows the farmer to get a return on investment. Solutions focus on increasing distribution channels that serve as the middle person getting the product from the local producer to retailors who interface with consumers [32,33]. In this discourse, the food chain narrative stops short of considering the economic and social conditions experienced by the consumers in communities in which the farmer operates. The production, distribution, and infrastructure discourse does not focus on building local supply chains that emphasize high rates of poverty experienced by rural consumers [34].

Food entrepreneur, West Virginia

Right now, the problem we’re solving now is how to make small farmers profitable across the state. How to maximize their efficiency. How to keep them from staying up at night worrying about certain things. So, I’ve believed for a long time that West Virginia has the capabilities to be an agricultural powerhouse in, you know, crop systems … the limitation we have is the abundance of flat land. But we don’t necessarily need that. We just need to be a little bit more creative in what we grow and how we grow. My biggest thing that I dislike is when people say local food should cost more. I think the local food should cost less … you increase the yield …

Community development specialist, West Virginia

… well, there are ways of thinking here and structural issues that mean that farmers—especially small, rural, you know, farmers could use a little bit of extra support to get into some of those opportunities. It’s [the value chain cluster initiative] a technical assistance program that was designed to help support food and farm businesses to expand their businesses by accessing capital through [name of fund] Investment Fund … So, the idea of the value chain is that, you know, farmers will get a fairer price for their goods and will be able to … create a livelihood based on that … I guess, kind of like equity … farmers are entering into contracts and sometimes the pricing’s not that great and they are really kind of controlled top down. And so, a value chain kind of ensures that the (indiscernible) [farmers] are taken into account and it’s an equitable situation …

### 3.2. Healthy, Organic, Local Food

This discourse links the production of food to the ideas of community, wholesomeness, and health. In this case, food is legitimate when it is acquired outside the modernized, “real” system of food production and supply [13,33]. This discourse affirms the worth of the food based upon where and how it is grown (i.e., locally, organically, hydroponic, microgreens, and/or pesticide-free). Failure to buy and consume food produced in the place prescribed and by one of these special methods represents acceptance of a less wholesome version of food. In this discourse, eating food serves a purpose beyond nutrition, where the community comes into existence and achieves virtue when its interests are bound up with the interests of the farmers. In this discourse, eating local food, which is considered wholesome, brings the community together in what is meant to feel like a collective act. The notion of health, unlike physiological health resulting from adequate nutrition, gives rise to a discourse that objectifies health as food that is local, organic, or produced and processed in any number of ways that excludes the modernized food system [13,33]. The discourse on local foods demonizes the modern food system as one that supports energy-dense and nutrient-poor “food,” especially for food-insecure people, and bestows superpowers on local food to reduce obesity and diabetes and increase household food security.

Food systems expert, West Virginia

A lot of my job is working across the state with local producers and how we aggregate local food into our systems and food access is a big concern of ours and of course why everyone is in this room … food access in West Virginia is obviously a very complicated situation there’s lots of factors at play [as to] why we are food insecure and a lot of these have to do not just with identified food deserts … so [video plays] “what West Virginia has is a lot of land we have the opportunity to create farms to help people create their own farms whether it’s in their backyard or if they have 100 acres [company] farm collective is a company that allows farmers to do what they do and that is farm that is grow crops what we provide is a service that markets their product, sales their product aggregates their product our goal is to stimulate the economy and bring healthy food options to this state our producers are accessible you know where your food came from and there really is something to be said for that we have an online farmer’s market that allows even the smallest farmer to list their product …

Food advocate, undergraduate student, West Virginia

I think now more than ever people care what is in their food and are asking more questions than ever before about what we are putting into our bodies.

Food academician, Arkansas

Modern farming practices are reducing the number of edible species of plants, animals, and therefore nutrients; Ensuring food safety from production to plate; “Progress” continues to subsidize unhealthy, nutrient-poor foods.

### 3.3. Behavioral Health and Education 

This discourse focuses on consumers and medicalizes the food system as nutrition, diet, and individual behavior with an emphasis on solutions that call for education and/or policing personal conduct [35]. This discourse minimizes or ignores the contribution of the non-medical determinants of health to population health, (i.e., transportation, income, or neighborhood conditions), eventually asserting individual responsibility as the basis for good or poor health [36,37,38]. One underlying message is that having poor health results from “deviant” behavior, where those who suffer from diet-related diseases and sickness have failed to exhibit a sufficient degree of personal responsibility for their lives and more specifically what foods they eat [39]. Solutions in this narrative emphasize that personal conduct and knowledge about nutrition, if regulated and enhanced, would solve poor health outcomes. Within this discourse, still failing to acknowledge non-medical determinants of health, the only other acceptable excuse for good versus bad food choices, falls back on socialization attributed to culture, family, or ethnicity [40,41]. Finally, in this narrative, food exists in service to the health of a body devoid of issues related to pleasure, appeal, social processes, and even temporal, geographical, and historical factors [36,37,42].

Surgeon, West Virginia

Well, as you know, obesity’s a huge problem in this state—and I do cancer surgery. Obesity is linked to diabetes, which is linked to cancers. There’s an increased risk of cancer in patients who are obese, and in patients who are diabetic, and certainly in both. So, it made sense to me to try to see if there were anything we could do to modify the patient’s behavior. But it would start with having access to healthy food. When we got the business school involved and they were going to look into these various things. But I thought we needed a psychologist involved to see how we could modify their behavior. Because it’s my belief that a lot of people even if you put healthy food in front of them, they wouldn’t eat it. Well, I thought we should address that first. Because if they’re not going to change their behavior, even if they have free healthy food, then it’s not going to work.

Land conservationist, West Virginia

… depends on how you want to define food desert, cause I think people have access. The biggest problem I find with people, if you watch what people buy once they get to a grocery store, they don’t buy the right things … I guess everybody’s concerned on, you know, having high quality food and things, but if you watch what people buy, and I’ve seen—watched them used their cards [SNAP or WIC] around here, they’re still able to buy alcohol, and they buy a lot of chips and a lot of sweets, but they don’t buy, you know, substantial food for themselves. You know, meats and vegetables. Hardly ever see very many people spend a lot of money on raw vegetables. If they do, it’s all processed food.

Food academician, Arkansas

So many individuals, as well as younger generations, have forgotten how to cook—how to prepare meals using ingredients and food components. Availability and access mean little if food preparation skills are absent. A second point: parents may see food as a struggle for children. That is, children may not “like” certain foods, many of which tend to be healthy (vegetables). Therefore, parents may choose not to spend money on vegetables because they will likely go to waste.

### 3.4. Sustainability

This discourse is like an empty bucket into which food system advocates and experts dump a cornucopia of societal goals including natural resources and the environment, animal welfare, safe food, and farm worker protection [43,44]. The greatest benefits are achieved, according to the sustainability narrative, when practices involved in food production operate under the principle of “do no harm” [45,46]. The discourse stresses that food should exist in service to more than just sustenance and/or pleasure. Instead, the narrative posits that food has its highest value when it is a solution to or does not impair pursuit of other societal interests [3]. One narrative criticizes sustainability for being overused to cover a broad range of issues [47]. Another critical claim is that corporations are said to have captured sustainability as a means of legitimizing and enhancing their image. Two other counter claims to this discourse call for cautious, incremental coordination, and collaboration across complex sectors and an economics narrative weighing the costs and benefits to achieve sustainable food production [48,49].

Food academician, North Carolina

… who works on farms? Migrant labor issues, poor wages. People who are growing food can’t afford it. So, what do you mean here by “communities” growing their own food. Who is doing the growing should be a question and who has access to the land? Who has lost land because of colonial/post-slavery racist land-grabbing practices? What impact does that have on the local food system?

Agricultural doctoral student, West Virginia

Sustainability, at least on my side, has been defined as a three-legged stool. Here, you have to have all three parts for it to make sense. And one of those is the financials, or the economics, which—gets left out of the conversation a lot, in my opinion. One of those is the environmental thing, which I feel like is what people target a lot. And then the other part of that is the social aspect. That’s part of the reason why—I mean, it’s a big concept.

Academic extension agent, West Virginia

It’s why people don’t eat veal much anymore. It’s why people just drew the line at some sort of genetic modification of things. And it’s why—after a while people get really pissed when you’re wasting all the water … And so that’s the social thing. And then I would like to expand it to say that it would also include folks thinking, maybe, it’s socially the thing to do is to buy local food for all the environmental reasons and for your damn neighbors.

Agricultural doctoral student, West Virginia

Advancements in the food system leads to innovative technologies, which can decrease jobs as well. Robotic picking machines to reduce labor costs and potential injuries, drones to scout fields to reduce the amount of pesticides needed.

Hydrological researcher, West Virginia

The proposed program [by providing comprehensive hydrological and climatological data sets] will help contextualize a wide variety of research questions (e.g., plant science, animal science, forestry, land management, agricultural production) starting with requisite hydrological baseline data. The monitoring is used to identify water quality at receiving waters and can be useful (for example) to identify if nutrients (fertilizers) are being washed off-field, if so, a farmer could adjust timing or quantity of fertilizers to reduce waste and operating costs (investment in fertilizers).

### 3.5. Finance and Investment

This discourse emphasizes food security through supply side strategies that encompass retail market solutions [50,51]. In this discourse, market failure is assigned blame for the problem of food insecurity. The image of a food desert is most often used to make claims about the problem that must be solved [52,53,54]. The narrative champions improved nutrition and food security through improved access to opportunities for food retail [51]. Moreover, while this narrative speaks to the quality of food and pricing within the retail environment, it does not call into question consumer behavior, but rather makes claims about the responsibility of the food retailer to carry healthy, affordable food [52,53,54,55,56,57]. An important narrative championed by the discourse is the ability to buy food in the same way as your neighbors through conventional points of distribution [58]. Solutions might include government action to lower barriers to entry for supermarkets, incentives for food retailers, or public-private partnerships that configure retail opportunities. The narrative claims that use of a retail market solution to improve diet is thought to lower the prevalence of obesity and improve fruit and vegetable intake [54,59]. Counter narratives, largely from economists, question the financing of grocery retail experiences in the face of consumer behavior that links poor health and food to shopping habits, eating behavior, and knowledge about nutrition [60,61]. The use of these counter narratives feeds into discourse that blames the dietary habits of low-wealth groups on their individual-level eating behavior or educational levels.

National community economic development specialist, Pennsylvania

I work at reinvestment fund…we are a community development financial institution [CDFI] essentially a mission driven nonprofit bank … in 2004 in partnership with a number of organizations across the state of [anonymous] we launched a program called [anonymous] that initiative was started because a room that was somewhat similar to this kind of room had identified that there were areas in the city of Philadelphia and Pittsburgh and throughout the state in rural communities in small towns that there was limited access to supermarkets … an earlier speaker mentioned a food desert map … we have a similar tool that we call the limited supermarket area assessment and the LSA has that … layer that shows you how far away from a grocery store you are but it’s sort of a business focus tool that takes into account the market potential…what the capacity is to build new stores … in addition to the need … we’re very excited in particular about the evolution of the conversation from grocery store as solution to food desert to a more comprehensive look at the food system … so smaller solutions like mobile markets or pop ups … those kinds of solutions might be a better fit for the community and so [we] want to be able to provide resources to all of those different things.

Community development specialist, West Virginia

… for example, we conducted like a financial food camp workshop and one of the groups that came to that was [anonymous]. And they’re in [anonymous] down in the coalfield and they have like a mobile market. So, they’re traveling around to different communities and delivering products directly in senior centers and things like that. So, they’re really trying to address that [food deserts]. So, you know, I see us as trying to address food deserts just indirectly because what we’re really trying to address is helping the entities [in the supply chain] that are doing that to be more financially—like trying to figure out how to keep afloat and keep doing what they’re doing.

Land conservationist, West Virginia

So yeah, there’s—Dollar General—like Dollar General market—or Dollar General stores are just popping up left and right. You know, you’re on these back roads and they’re just in the middle of nowhere. It’d be great if they would be able to incorporate somehow like the fresh food. ‘Cause right now, they do have, you know, milk and eggs, but other than that, it’s pretty much all highly processed foods there. And that’s what people are turning to, because it’s convenient. They could just stop there, pick things up. And they, I think, are eating what’s there, because it’s easily available. Those types of stores—even, you know, your small like gas stations—I’ve noticed some of them have like apples and bananas and stuff, you know, oranges to grab, which is really nice to see, because it doesn’t—it does give them that access, rather than picking up a candy bar.

### 3.6. Hunger Relief

This discourse pits the food system of plenty against the problem of hunger, framing the battle as an issue of equity and economic justice for poor people [17,31,62,63]. The narrative adopts a critical analytical approach to contravene hunger relief discourses that fail to engage around action to solve the issue of poverty which is asserted as the ultimate cause of hunger [17,63,64]. Other hunger discourses that mask poverty relief narratives include sustainability, health, food waste, charitable food assistance, agricultural subsidies, and community–corporate relations. The hunger relief discourse asserts that these narratives only serve to mask ideas concerning economic policies, societal responsibility for the poor, and poverty [30,31,63]. For example, one narrative questions the positive value of the use of food waste and food charities to address hungers [62,65]. The hunger discourse calls into question narratives built around the idea that addressing food insecurity with food waste is an unassailable good [17,61,65]. The food waste passage below is an example of the narratives that make it harder for hunger discourse to take up the problem of poverty. In the passage below, the benefits of charitable and federal food assistance programs are touted as helpful initiatives that rescue food waste, help corporations be responsible citizens, and subsidize the agri-food industry by moving food where there is hunger without taking into the account the type of food, reasons for food scarcity, and the poverty that drives food insecurity.

Land conservationist, West Virginia

… something that we started in our community of Brooke County was like a gleaning project where, you know, if farmers had leftover fruits and vegetables, rather than throwing them in the compost pile or throwing them to livestock, we would donate those to a community, and that comes back to, you know, decreasing food waste which would all go together some way or the other.

Food systems, geography academician, West Virginia

The local canned food drive that still occupies much of the social imaginary surrounding food charity in the United States is implicated in a powerful network of food system actors that contribute to reproduce hunger under capitalism. Although food banks and their LFCs [local food charities] are largely perceived as organizations whose raison-d’être is to feed the poor, my institutional ethnography of the HFN [Hunger Food Network] in West Virginia complicates this idea. My analysis has demonstrated that the roll-out of the food bank fix is a fraught and contested part of a moral economy legitimizing the corporate food regime [58].

### 3.7. Demand-Side Preferences

This discourse explores the food system needs and preferences of low-wealth rural groups. The narrative exerts the primacy or role of consumer choice in the food system chain [16]. The discourse presumes that groups near the bottom of the social gradient will first and foremost be concerned with availability, accessibility, affordability, acceptability, and accommodation (Table 3) [38,66]. The narrative in this discourse argues against the idea that the health problems of low-wealth groups are caused by or solely attributable to poor eating behavior, lack of nutritional knowledge, or irresponsible behavior [67,68,69]. Narratives that push back against the individual responsibility trope include structuralist and social determinants of health theories that attribute poverty and poor health to forces outside the individual including transportation, poverty, wages, the regional economy, and employment [36,38,70,71,72,73,74,75,76].

Community organizer, West Virginia

I have never heard an impacted person mention jobs and how that plays into the food accessibility problem. There also hasn’t been a lot of talk about where the food comes from when purchasing it from the store. Remember that a lot of folks don’t have the privilege of food choice, so where it comes from or who is employed through the food system isn’t really a concern. It’s cheaper to buy 10 boxes of Hamburger Helper when they’re on sale 10/$10 rather than a bag of oranges that costs $7. With that being said, I believe the conversations could be steered toward economics and workforce development, but I would be surprised if it popped up organically.

### 3.8. Romanticized, Community Led Transformations

This discourse rests upon a romanticized, rose-colored vision that seems to have not integrated the needs and preferences of groups of a different social status from the food experts [77,78,79,80]. In this case, the expert discourse envisions empowered, politically savvy, organized low-wealth groups who desire a local food system and are eager to secure food through traditional means that eschew the desire for supply side retail options. In addition to asserting the interest of low-wealth rural groups in local food systems, the discourse also asserts that communities are self-reliant and capable of (re)building a communal food system [3]. In this discourse, communities are portrayed as a cohesive group that comes equipped with a collective desire to take action along with an intact level of civic capacity expressed as a sense of shared values and the ability to undertake critical reflection, dialogue, and problem solving along with mechanisms by which to engage each other and those in power [3,81]. The communal food system is made manifest in how communities acquire food through gardening, hunting, or exchanges and bartering between neighbors. For example, one narrative put forth by food experts and advocates frames foraging as a practical, reliable, everyday way that rural families might use to meet their needs and/or as an appropriate way to supplement food comparable to the way that other families around them shop for food.

Solutions in this discourse are presented as an empowered collective group, community led entrepreneurial activities that provide jobs, revitalization of traditional ways of securing food, and/or a communal food system. When an equity narrative on power imbalances is inserted these solutions come closer to representing community led initiatives that can address structural inequity [77,78,79]. At other times, the romanticized discourse does not take into account that low-wealth rural families may not desire a local food system, have time or aspirations for foraging or gardening, or may want an experience that is similar to other families around them that have the resources to secure food. Finally, this discourse ignores that these families most likely have little or no civic capacity and live within social systems that are broken because of historical and political forces that lie outside the control of low-wealth rural communities [79,80,81,82,83,84].

Take Action Website

Start with understanding your community. Gather data on demographics, diet-related diseases, regional food production and access to healthy food. Also look at existing food projects in your community. You might partner with the city, university or college, or a nonprofit organization to help collect data. Equally important is listening to the community-what are people saying about needs and priorities. Assess what you’re hearing, along with the data to identify potential gaps and opportunities [85].

Extension Agent, West Virginia

… as individual pleasure, I would include family traditions, culture, food for special occasions. Under farming, I might include traditions, too. I have heard people talk about planting during a full moon or other such folklore.

Government conservationist, West Virginia

I think for one is, you know, back in the day, even if you necessarily didn’t maybe grow or produce your own food, your neighbor did. There was like—they would kind of, you know, trade or barter things. Now the society really does rely on going to their store for food. To me, that’s a big concern, is there’s no connection between how the food gets on people’s tables.

Food academician, North Carolina

I think that the legacy of farming for rural areas goes beyond just that they can grow food, there’s also a pride and a sense of identity associated with farming … a sense of connection with other people—family traditions, shared meals, the importance of passing down recipes; food preservation as a tradition and part of rural culture and life…

## 4. Discussion

This analysis identified eight aspirational food system discourses curated by food system experts and advocates. These dominant food system discourses were presented along with narrative profiles and exemplary passages extracted from the analysis of the qualitative data (Table 3). Of those eight discourses, three (finance and investment, hunger relief, and demand-side preferences) demonstrated narratives and claims that represent the lived experiences, preferences, and needs of low-wealth rural residents. For example, this study demonstrates how the demand-side discourse represents the lived experiences of low-wealth rural groups by problematizing poor nutrition as an issue of access. This discourse not only illustrates the needs of low-wealth rural groups but, in recent research, has been positive in shifting responsibility for a poor diet from the consumer’s behavior to the issue of food availability [86,87,88]. In comparison, this study also demonstrates how other discourses increase the risk of inequities through the assertion of dominant, aspirational food goals that serve to silence or silence and make hard to hear discourses that represent less influential or powerful groups such as low-wealth rural residents [88,89].

For example, one industry report generated by the co-authors of this paper detailed the lived experiences of 153 low-wealth rural individuals in such a way as to illustrate the unheard claims around structural causes of food insecurity related to poverty, food preferences, and how to address or not address needs and preferences of rural groups living in poverty [90]. Passages below from the interviews illustrate these less frequently heard claims.

### 4.1. Structural Causes Income, Poverty, Transportation

North Carolina

It was to the point where I was going from either hotel to hotel or when money got low, then, I was trying to save the money for the food and we were staying in our vehicle.

I think this is probably like the poorest county in North Carolina. I think I heard that somewhere. So, I mean, that plays a part in a lot of things … [T]here’s not much going on for the kids and not much going on as far as like transportation and stuff. A lot of people around here need transportation.

It’s not no opportunities here. It’s hard to get a job here. Like nothing. … If you want to succeed you have to go to college and do something. And we’re in an epidemic right now for heroin and fentanyl.

Not any jobs. The jobs that we do have is mostly restaurants. We need more higher paying jobs.

### 4.2. Role of Corporations in Poor Nutrition

Montana

We don’t have a lot of the best foods, it’s really hard to buy fruit and vegetables and fresh produce all the time. You just can’t. And so pretty much everything’s processed. But we survive.

There’s no need for pop to be bought with food stamps. It’s—costs our health care millions of dollars and it’s not good. Tastes good, but it’s no nutrition, no value at all, and it’s—all it is is bad for you.

North Carolina

Yeah, maybe I just need to just find me some water and just chill out, but I really think that that’s where some of these cancers and stuff is coming from, is from all these processed foods.

I don’t eat McDonald’s no more. […] I was watching a You Tube video and they said something about McDonald’s food don’t break down, I don’t know, I was scared about that.

### 4.3. Possible Solutions

North Carolina

The Dollar Tree-little like seasonings and stuff like that, you know, stuff like that ain’t nothing but a dollar. And snacks for him—they have little packages for a dollar. I go in there and get them for him.

Texas

There needs to be more opportunities and more groceries. Like I was telling you, we only have a Walmart that does not have groceries and then you have the two other grocery stores that have everything like expensive.

West Virginia

I don’t know about anybody else, but sometimes like the only grocery store we have in town their prices are kind of high, I think. And that’s why sometimes I choose to go to the Dollar Store then what I would at a regular grocery store because the prices are too high.

### 4.4. Preferences

North Carolina

I just love I guess a good old Sunday dinner. I like, like when I cook, I like cabbages, and macaroni and cheese is one of the main dishes when I cook a dinner or something. Macaroni and cheese. Maybe fried chicken. … I always try to cook a green, if not, some French style green beans. I like those.

West Virginia

[One of the food pantries] [The local food pantry, they] have a [community] truck day and they give desserts and things for the kids too, or like fresh fruit.

[My ideal meal is] Something balanced. You know, you can’t just have one item. You gotta have at least one meat and a couple sides and a bread.

Like TV dinners, the little hamburgers, pancakes, and stuff like that. They love vegetables. They eat about anything.

It would seem from the quotes that, low-wealth rural groups have preferences and needs that are markedly different from the aspirational vision curated by food experts and advocates, focusing instead on income and what is available. Other claims from low-wealth rural groups wonder why and how corporations contribute to poor nutrition and question the lack of strategies and policies to address transportation, poverty, and unemployment. On the whole, it would seem that rural groups focus on practicalities such as the five A’s of food access (Table 4) [37,66].

The analysis in this paper illustrated how food experts and advocates have curated a food vision that authenticates an aspirational image of food that ignores the discourses and preferences of low-wealth rural groups. We presented three silenced and less frequently heard discourses on the role of poverty, the demand for retail configurations, and political and economic power of the corporate and business sectors in the inequitable distribution of income all overshadowed by five discourses that view food and the food system as a set of aspirational goals that, if one chooses wisely, bestows social status and delivers a wide range of outcomes including good dietary habits, local community economic development, a better environment, community power, and reduced food waste.

Overall, this discourse analysis lends credibility to the idea that rhetoric, words, and ideas might be taken to constitute not only reality, but actively constitute systems of power that dictate the selective preservation of knowledge that is then taken as “truth” [17,31,91,92]. In the future, it is important to explore further how these aspirational visions contradict—and may even harm—the low-wealth rural communities they aim to serve because they are rooted in normative ideas about a definition of “good” that professes to be universal, but in many cases reflects differences in groups based on social status arranged along a gradient from top to bottom where those in the middle and at the top get to determine what is acceptable [93,94,95]. More work needs to be performed around whose voices are being heard and what voices are being suppressed so that public policy, programming, and food systems work will not continue to perpetuate this misfit between low-wealth rural communities and the “experts” who seek to serve them.

## 5. Conclusions

While discourses do not typically reveal the truth or falseness of ideas, discourse analysis can help to discern who is empowered, what voices are dominant, and who is shut out [12]. Findings from this study on the aspirational discourses on food systems curated by food experts and advocates revealed that of eight discourses, only three encompassed the lived experiences of low-wealth rural residents. This aspirational food system, if not tempered, may result in: (1) suppression of the needs and preferences of low-wealth rural groups, (2) a perpetuation of inequality between groups who can and cannot attain the aspirational food vision, (3) the silencing of discourses that might question the political and economic power of corporate and business sectors that play a role in the inequitable distribution of income while sanctioning discourses that focus on personal or community solutions; and (4) the absence of other policy-based solutions that might address issues located within the food system as opposed to those with people and their diets and personal characteristics [17,31]. To close the gap between low-wealth rural communities and the “experts” who seek to serve them, experts should strive to draw attention to less dominant discourses that represent the needs and preferences of low-wealth rural populations if they aim to tailor policies to address poor diets caused by food insecurity.

## Figures and Tables

**Table 1:** Food System Expert Demographics.

	Type	Number (%)
Sector		
	Academic	18 (64.3%)
	Community Advocate	4 (14.3%)
	Private Sector	2 (7.1%)
	Funder	1 (3.6%)
	Community Foundation/Philanthropy	1 (3.6%)
	State Government	1 (3.6%)
	Academic and Community Advocate	1 (3.6%)
Affiliated with a University		
	Yes	20 (71.4%)
	No	8 (28.6%)
Years of Experience in Food Systems		
	Less than 5 Years	16 (57.1%)
	6 to 14 Years	7 (25%)
	15 or More Years	5 (17.9%)
Region		
	Northeast	16 (57.1%)
	Southeast	7 (25%)
	Central	4 (14.3%)
	West	1 (3.6%)
Gender		
	Female	20 (71.4%)
	Male	8 (28.6%)
	Non-Binary/Third Gender	0 (0.0%)
Race/Ethnic Background		
	White	23 (82.1%)
	Black or African American	5 (17.9%)
	American Indian or Alaskan Native	2 (7.1%)
	Asian	0 (0.0%)
	Native Hawaiian	0 (0.0%)
	Other Pacific Islander	0 (0.0%)
Hispanic, Latino/a, or Spanish Origin		
	No	27 (96.4%)
	Yes	1 (3.6%)

**Table 2:** Screening Questions for Narrative Profiles.

Screening Question	Components
Big idea, knowledge preserved.	Common narratives, themes, terms, phrases.
How are these discourses illustrative of the producer’s understanding of the world?	What is causing the problem? What are the solutions?
What cannot be said?	Who may (not) speak with authority? What or who is silenced? What discourses become “bad”, less acceptable, under used, not heard?

**Table 3:** Thick Descriptions of Discourses and Narrative Profiles.

**Discourse**	**Big Idea, Knowledge Preserved**	**Discourse Producer’s Understanding**	**What Cannot Be Said**
Romanticized, Community Led Transformations	The community is a cohesive collective that desires and is capable of creating a local food system that includes their traditions and embraces the production of food that upholds ethical ideals such as equity, sustainability, and health.	Problem is modern day food system that is out of reach, impervious to community wishes, and reduces reliance on traditions; solution is to build an empowered community that wants to use its traditional methods of food production.	Low-wealth groups may have other priorities/needs, prefer other kinds of food or convenience; have limited incomes and time that compete with building a food system.
Demand-Side Preferences	Groups near the bottom of the social gradient are concerned with whether food is available and how to get that food (location, affordability), whether the food fits their preferences, and how they are regarded by others (vendors, etc.).	Problem is narratives that present low-wealth groups as responsible for their poor nutrition and bad health, i.e., uneducated, low/no motivation; solutions are to present counter narratives that illustrate structuralist explanations for eating behavior like food security, cost, availability, accommodation, accessibility, transportation, social determinants of health, the chain of events and circumstances over the life course that perpetuate the cycle of poverty.	Groups near the bottom of the social gradient do not buy the right food, eat well, and many times have no cooking skills.
**Discourse**	**Big Idea**	**Producer’s Understanding**	**What Cannot Be Said or Heard**
Hunger Relief	Hunger relief has been captured by institutional interests that overshadow structural conditions and policies that can alleviate hunger by addressing income inequality and poverty.	Problem is other narratives on food waste, corporate giving, etc., that ignore economic drivers that cause hunger; solutions include hunger relief policies that address income equality and poverty.	Charitable and federal food assistance programs are helpful initiatives that rescue food waste, help corporations be responsible citizens, and subsidize the agri-food industry by moving food where there is hunger.
Finance and Investment	Market failure is to blame for the problem of food insecurity.	Food deserts and the lack of sources of affordable, healthy food are the problem causing food insecurity. Solutions focus on strategies that increase retail development in places that are food deserts.	Low-wealth groups may not improve their eating habits even with the presence of affordable shopping experiences. They may need help with shopping decisions, knowledge about nutrition, cooking, and eating behavior.
Sustainability	Food production is an instrument for transgressions towards any number of moral ideals that cover economic, social, and environmental practices including natural resources and the environment, animal welfare, safe food, and farm worker protection.	The problem is large scale food production practices and supply chains that seek profits over humanitarian and environmental goals. Solutions focus on using sustainability to force institutions and systems to confront and institutionalize structural drivers of their unsustainable practices.	Sustainability has become a trope with no specific meaning. Incremental action, the costs and benefits, and tradeoffs must be considered when trying to enforce these big moral ideals.
Behavioral Health and Education	The food system is a medical tool that delivers nutrition and those who choose to select the right food have the best chance of good health.	The problem is that groups that experience the greatest rates of poor health do not know how or do not care about behaving properly to achieve good health. Solutions emphasize education, penalties or incentives for groups that behave responsibly.	Food choices are complex depending on a range of issues that blend together including socialization but also structural factors as well including income, geography, and transportation.
Production, Distribution, and Infrastructure	A robust network is needed to grow, process, distribute, and market products so that smaller farmers can earn an income. The narratives usually end with a food chain that concludes when the farmer’s products are bought by a distributor at fair market value that allows the famer to get a return on investment.	Problem is the lack of a food supply chain that provides enough food access points (venues) that sell the producer’s local products. Solution is to establish more business arrangements that allow the producers to sell their products by connecting them to better health outcomes or economic development.	Producers need to build supply chains grounded in equity that covers their needs while taking into account the economic and social conditions present in the communities in which they operate.
Healthy, Organic, Local Food	Food is legitimate when it is acquired from local farmers/producers and outside the modernized, “real” system of food production and supply; it is anti community to buy and eat food outside the local community and represents acceptance of a less wholesome version of food.	The problem is the inability of local producers to be profitable when a giant modern food system makes it easy, cheap, and faux healthy to buy and consume their food. Solutions include educating the public about food and health and increasing systems and venues so that the public will be able to acquire local food.	In many rural places, people cannot afford, do not have time, and prefer other foods in order to maximize their resources and time that must be spent in pursuit of other important needs.

**Table 4:** Five A’s of Food Access.

Five A’s	Definition
Accommodating	Are vendors and retailers aware of consumers’ needs?
Acceptable	Does the food fit the taste and culture of consumers?
Available	Is the food present?
Accessible	Can consumers get to it?
Affordable	Can consumers afford it?
